# Systematic Review and Meta-Analysis of the Relationship between *EPHX1* Polymorphisms and the Risk of Head and Neck Cancer

**DOI:** 10.1371/journal.pone.0123347

**Published:** 2015-04-29

**Authors:** Hong Chen, Lin Ge, Qiuli Sui, Mei Lin

**Affiliations:** 1 State Key Laboratory of Oral Diseases, Sichuan University, Chengdu, Sichuan, PR China; 2 Department of Oral medicine, West China School of Stomatology, Sichuan University, Chengdu, Sichuan, PR China; 3 Department of Oral medicine, West China School of Stomatology, Sichuan University, Chengdu, Sichuan, PR China; Kyushu University Faculty of Medical Science, JAPAN

## Abstract

**Aim:**

To evaluate the association between the *EPHX1 Tyr113His* and *His139Arg* polymorphisms in the *EPHX1* gene and the risk of head and neck cancer.

**Materials and Methods:**

Studies on the association of *EPHX1 Tyr113His* and *His139Arg* polymorphisms with HNC performed up until June 1st, 2014, were identified using a predefined search strategy. Summary odds ratios (ORs) and 95% confidence intervals (CIs) were used to evaluate the strength of these associations.

**Results:**

In this meta-analysis, 10 case-control studies, which included 9 studies of *Tyr113His* (1890 cases and 1894 controls) and 10 studies of *His139Arg* polymorphisms (1982 cases and 2024 controls), were considered eligible for inclusion. Overall, the pooled results indicated that the *EPHX1 Tyr113His* polymorphism was significantly associated with increased HNC risk (*Tyr/His* vs. *Tyr/Tyr*, OR = 1.26, 95%1.02–1.57;*His/His*+ *Tyr/His* vs. *Tyr/Tyr*, OR = 1.29, 95% I = 1.03–1.61). However, no significant association was found between the *His139Arg* polymorphism and HNC risk. In the subgroup analysis, a statistically significant association between the *EPHX1 Tyr113His* polymorphism and HNC was observed in population-based case-control studies (PCC), which involved less than 500 participants and genotype frequencies in HWE. This association showed minimal heterogeneity after excluding studies that were determined to contribute to heterogeneity. After categorizing the studies by publication time, a sensitivity analysis and cumulative meta-analysis of the two associations were conducted, and the results of the two analyses were consistent.

**Conclusion:**

Our meta-analysis suggests that *EPHX1 Tyr113His* polymorphism may be a risk factor for HNC, while the *EPHX1 His139Arg* polymorphism has no association with HNC risk.

## Introduction

Head and neck cancer (HNC) is the fifth most common type of human cancer [1], with approximately 434,000 new cases diagnosed annually worldwide [2]. HNC occurs more frequently in developing countries, such as India, Brazil, and Thailand [3–4]. Although the mechanism underlying HNC is still not fully understood, accumulating evidence suggests that tobacco smoking, drinking alcohol, and chewing betel quid are three main risk factors for HNC [5–6]. Several previous studies reported that tobacco smoke and alcohol metabolites may induce defects in DNA and genomic instability, which can lead to mutations and malignant transformation [7–9].

Microsomal epoxide hydrolase (*mEH*) (*EPHX1*), which is one of the phase I detoxification enzymes found on the endoplasmic reticulum in many tissues, plays a role in the metabolism of potential carcinogens in tobacco smoke[10]. Epoxides, with their highly reactive oxidative metabolites, are often the most toxicologically active drugs and environmental chemical [11]. *EPHX1* catalyzes the hydrolysis of reactive epoxides to trans-dihydrodiols, and some dihydrodiols can be subsequently metabolized to highly mutagenic polycyclic hydrocarbon diol epoxides [12–13]. Therefore, *EPHX1* plays a dual role in the activation and detoxification of procarcinogens. Moreover, some studies have reported that the role of *EPHX1* in carcinogenesis may depend on exposure to different environmental substrates [14].

The *EPHX1* gene is 35.48 kb in length with nine exons and eight introns [11], and it is located on chromosome 1q42.1. More than 110 single nucleotide polymorphisms (SNPs) have been identified in the *EPHX1* gene, and these *SNPs* can be found in the dbSNP database (http://www.ncbi.nlm.nih.gov/SNP). Two alleles of *EPHX1* are common and have been associated with altered enzyme activity [15]. With the differential effect of *EPHX1* alleles on the detoxification of procarcinogens, we proposed that the two functional polymorphisms may affect HNC risk.

Several previous studies were conducted to evaluate the association between *EPHX1* polymorphisms (*Tyr113His and His139Arg*) and the risk of HNC in different populations; however, the results of these studies were inconclusive. Up to now, no remarkable evidence has been presented to suggest the precise role of *EPHX1* (*Tyr113His and His139Arg*) in HNC. Accordingly, a comprehensive evaluation of the associations between *EPHX1* polymorphisms (*Tyr113His and His139Arg*) and HNC risk is urgently needed. In this study, we reviewed the existing literature and performed a meta-analysis to evaluate the association between *Tyr113His* and *His139Arg* polymorphisms in the *EPHX1* gene and HNC susceptibility.

## Materials and Methods

### Search strategy

We conducted a computerized literature search of Medline, PubMed, EMBASE and China National Knowledge Infrastructure Whole Article Database (CNKI) prior to the June 1st, 2014 using the following key words: (‘microsomal epoxide hydrolase’ OR ‘*EPHX1*’ OR ‘*mEH*’) AND (‘head and neck’ OR ‘oral’ or ‘oropharynx’ OR ‘larynx’ OR ‘pharynx’) AND (‘cancer’ OR ‘tumor’ OR ‘carcinoma’) AND (‘polymorphism’ OR ‘variant’ OR ‘allele’ OR ‘genotype’). The search was not restricted by language but was restricted to human studies. The references cited by the identified original studies and relevant reviews were searched at the same time. Furthermore, we contacted some experts in the area to obtain information about unpublished results or ongoing trials nearing completion.

### Inclusion and exclusion criteria

Relevant articles and abstracts were selected and reviewed independently by two of the authors (Hong Chen and Lin Ge). The following inclusion criteria were used for published studies: (i) case control studies that were performed to assess the association between at least one of the two polymorphisms (*Tyr113His* and/or *His139Arg*) and HNC risk; (ii) papers that clearly describe HNC diagnoses and the sources of cases and controls; (iii) papers that include sufficient genotype data such that the odds ratios (ORs) and 95% confidence intervals (CIs) can be calculated. If the samples described in two studies overlapped, we selected the study in which the larger sample was identified. The major exclusion criteria were as follows: (i) papers classified as reviews, editorials, letters, comments, or case reports; (ii) cell line studies; (iii) duplicate studies; (iv) studies in which the participants had distant metastasis or synchronous malignancy in other organs.

### Data extraction

Working independently, two authors (Hong Chen and Lin Ge) extracted the data from all eligible publications according to the inclusion criteria listed above. Any disagreements in data quality scores and abstraction were assessed further and dealt with by discussion between the authors. The following characteristics were extracted: the first author, year of publication, country of origin of participants, ethnicity, cancer type, source of control group (population- or hospital-based controls), number of cases and controls, genotypes, minor allele frequency (MAF) in controls, and P for Hardy–Weinberg equilibrium (HWE) (Table 1). In this meta-analysis, we defined a population-based case-control study (PCC) as a study that enrolled controls from healthy populations, and we defined a hospital-based case-control study (HCC) as a study that enrolled controls from hospitalized patients, as reported in previous studies [11,16].

**Table 1:** Characteristics of studies included in this meta-analysis.

Author	Year	Country	Ethnicity	Cancer types	*SNP*s studied	Source of Control	Sample size (case/control)	Specimen	Genotyping Methods	MAF in Controls	P for HWE
Jourenkova-Mironova	2000	France	Caucasian	oral/pharyngeal/laryngeal cancer	*Tyr113His*; *His139Arg*	HCC	250/172	peripheral blood	ASO-PCR	0.40;0.14	0.36;0.22
Amador	2002	India	mixed	oral/pharyngeal/laryngeal cancer	*Tyr113His*; *His139Arg*	PCC	137/99	peripheral blood	RFLP-PCR	0.45;0.16	0.69;0.05
Lacko	2008	Netherlands	Caucasian	oral/pharyngeal/laryngeal cancer	*Tyr113His*; *His139Arg*	PCC	429/419	peripheral blood	RFLP-PCR	0.31;0.20	0.55;0.70
To-Figueras	2002	Spain	Caucasian	laryngeal cancer	*Tyr113His*; *His139Arg*	PCC	204/203	peripheral blood	RFLP-PCR	0.31;0.19	0.24;0.96
Wenghoefer	2003	German	Caucasian	oral/pharyngeal/laryngeal cancer	*Tyr113His*; *His139Arg*	PCC	280/289	peripheral blood	Taqman	0.31;0.22	0.51;0.13
Boccia	2008	Italy	Caucasian	oral/pharyngeal/laryngeal cancer	*Tyr113His*; *His139Arg*	HCC	210/245	peripheral blood	RFLP-PCR	0.28;0.20	0.01;0.10
Balaji	2011	India	Asian	oral cancer	*Tyr113His*; *His139Arg*	PCC	157/132	peripheral blood	Taqman	0.39;0.23	0.80;0.37
Varela-Lema	2008	Chile	Caucasian	oral/pharyngeal cancer	*His139Arg*	HCC	92/130	peripheral blood	RFLP-PCR	0.17	0.10
Park	2003	USA	Caucasian	oral/laryngeal cancer	*Tyr113His*; *His139Arg*	HCC	143/213	either buccal cells or tissue	RFLP-PCR	0.38;0.18	5.56E-09;0.91
	2003	USA	African American	oral/laryngeal cancer	*Tyr113His*; *His139Arg*	HCC	80/122	either buccal cells or tissue	RFLP-PCR	0.18;0.30	0.00;0.74

**Abbreviations**: *SNP*s: single nucleotide polymorphisms; HCC, hospital-based case-control; PCC, population-based case-control; PCR-RFLP, polymerase chain polymorphism reaction-restriction fragment length; ASO-PCR, allelespecific oligonucleotide-polymerase chain reaction; MAF, minor allele frequency; HWE, Hardy–Weinberg equilibrium.

### Data synthesis

All data from the eligible studies were abstracted. We first evaluated HWE in the controls for each eligible study using the chi-square goodness of fit test, and P>0.05 was considered statistically significant. Crude odds ratios (ORs) and 95% confidence intervals (95% CIs) were used to estimate the strength of the association between HNC and the *EPHX1 Tyr113His* and *His139Arg* polymorphisms. A Z-test was also used, and P _values_ <0.05 indicated statistically significant associations. Pooled ORs were estimated for genetic models including the dominant model, recessive model, homozygote comparison and heterozygote comparison. Using *EPHX1 Tyr113His* as an example, the codominant model would be *His/His* vs. *Tyr/Tyr* and *Tyr/His* vs. *Tyr/Tyr*, the dominant model would be *His/His*+ *Tyr/His* vs. *Tyr/Tyr*, and the recessive model would be *His/His* vs. *Tyr/His*+ *Tyr/Tyr* [17].

We tested statistical heterogeneity by using Cochran’s Q statistic [18] and the I^2^ statistic [19], where *P*<0.1 was considered significant heterogeneity, and I^2^>50% indicated large heterogeneity. If heterogeneity existed, a random-effects model (the DerSimonian and Laird method) was adopted [20]; otherwise, a fixed-effects model (the Mantel-Haenszel method) was adopted [21] as appropriate. In addition, heterogeneity was also examined in subgroup analyses by ethnicity, source of controls (HCC/PCC), study sample size (≥500/<500 subjects), matched control (Yes/No), and HWE in controls (Yes/No). The reliability of the results was evaluated by performing sensitivity analysis. A funnel plot, Begg’s rank correlation method [22] and the Egger’s weighted regression method [23] were adopted to statistically assess publication bias (*P* < 0.05 was considered statistically significant). All analyses were performed using STATA software, version 12.0 (STATA Corp., College Station, TX, USA).

## Results

### Description of the included trials

**Fig 1:**
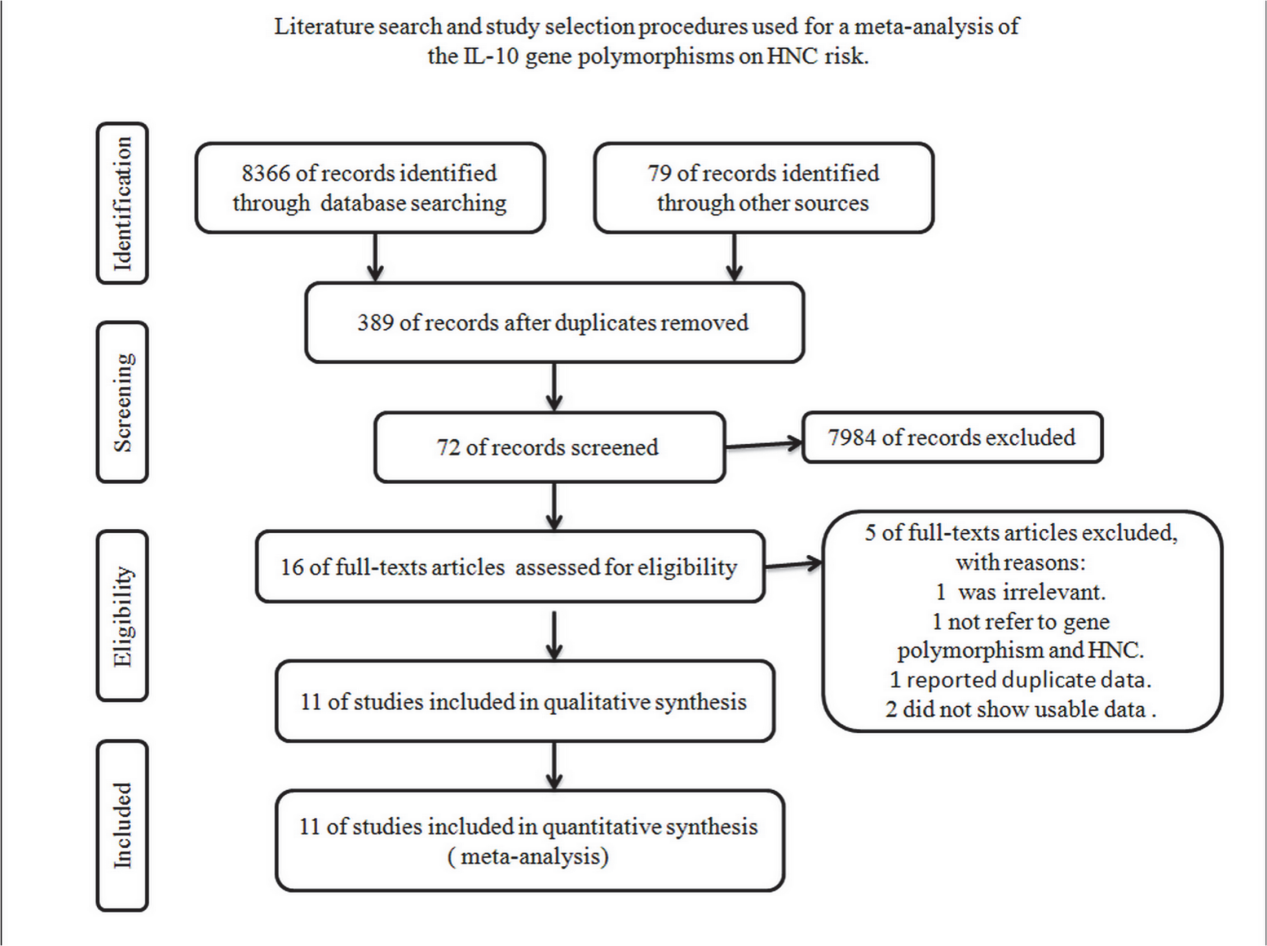
Flow chart of study selection and specific reasons for exclusion from the meta-analysis.

267 publications relevant to the search words were identified, and all of them were written in English. The two reviewers (Hong Chen and Lin Ge) independently screened the title and abstract with the focus question, and12 articles were identified [24–35]. Of these articles, three were excluded after full-text assessment for eligibility for the following reasons: one study was irrelevant, one study did not examine the association between these two polymorphisms (*Tyr113His* and *His139Arg*), and one study (an HNC risk study) did not yield an accurate result. A flow chart illustrating the study selection and specific reasons for exclusion is presented in Fig 1. Thus, 10 studies reported in 9 articles [24–32], which included 9 studies of *Tyr113His* (1890cases and 1894 controls) and 10 studies of *His139Arg* polymorphisms (1982 cases and 2024 controls), were found to match our inclusion criteria. One article [31] mentioned two independent case-control studies (Caucasians and African Americans), and the article was thus treated as two separate estimates.

The characteristics of the studies included in the meta-analysis are presented in Table 1. The ethnicities studied included Asians, African Americans, Caucasians, and mixed ethnicities. The studies were carried out in India, the Netherlands, Spain, Germany, Italy, Chile, France, and the USA. Among these studies, 5 studies focused on oral/pharyngeal/laryngeal cancer, 3 on oral /laryngeal cancer, 1 on oral cancer and 1 on laryngeal cancer. Most of the studies involved extraction of DNA from peripheral blood and employed the classic PCR-RFLP assay and PCR for genotyping. The genotype distributions among the controls of all studies followed HWE except for two studies [28, 31] that examined the *Tyr113His* polymorphism.

### Quantitative synthesis

#### The *EPHX1 Tyr113His* polymorphism and HNC susceptibility

**Fig 2:**
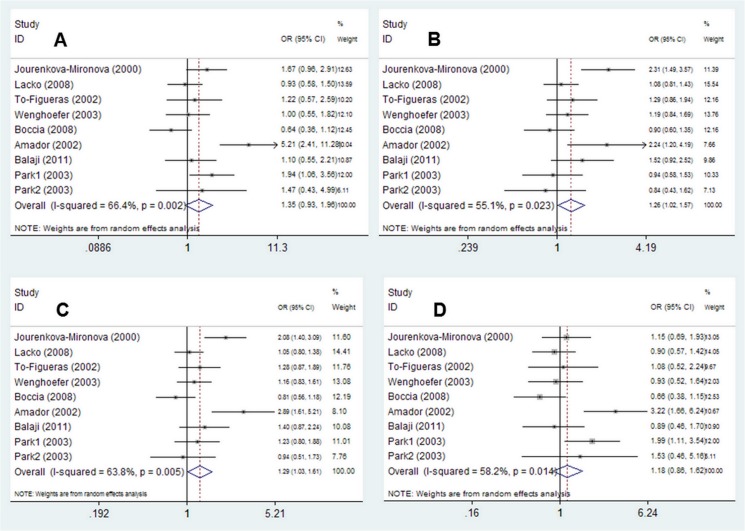
Forest plots of ORs with 95% CIs for *EPHX1 Tyr113His* polymorphisms and HNC risk. The center of each square represents the OR, the area of the square is the number of sample and thus the weight used in the meta-analysis, and the horizontal line indicates the 95%CI. (A) *Tyr/His* vs. *Tyr/Tyr*. (B) *Tyr/His* vs. *Tyr/Tyr*. (C) *His/His*+ *Tyr/His* vs. *Tyr/Tyr*. (D) *His/His* vs. *Tyr/His* +*Tyr/Tyr*.

Eight articles [24–29, 31–32] included in this meta-analysis described 9 case-control studies, with 1890 cases and 1894 controls, revealing an association between the *EPHX1 Tyr113His* polymorphism and HNC susceptibility. The main results of this pooled analysis are presented in Table 2 and Fig 2 shows forest plots illustrating the effect of the *EPHX1 Tyr113His* polymorphism on HNC risk. Overall, the combined results based on all studies showed that the *Tyr113His* polymorphism was significantly associated with HNC susceptibility (homozygote comparison model, *Tyr/His* vs. *Tyr/Tyr*, OR = 1.26, 95% 1.02–1.57; dominant model: *His/His*+ *Tyr/His* vs. *Tyr/Tyr*, OR = 1.29, 95% I = 1.03–1.61). However, the same association was not found in the homozygote comparison or recessive genetic models (homozygote comparison model, OR = 1.35, 95% CI = 0.93–1.96; recessive model, OR = 1.18, 95% CI = 0.86–1.62).

**Table 2:** Quantitative analyses of the *EPHX1 Tyr113His* polymorphism on the head and neck cancer risk.

Genetic model			Homozygote		Heterozygote		Dominant model		Recessive model	
Variables	Sample size		*His/His* vs. *Tyr/Tyr*		*Tyr/His* vs. *Tyr/Tyr*		*His/His*+ *Tyr/His* vs. *Tyr/Tyr*		*His/His* vs. *Tyr/His* +*Tyr/Tyr*	
	N^a^	Case/control	OR(95%CI)	*P*_*value*_ ^b^	OR(95%CI)	*P*_*value*_ ^b^	OR(95%CI)	*P*_*value*_ ^b^	OR(95%CI)	*P*_*value*_ ^b^
Total	9	1890/1894	1.35(0.93,1.96)	0.00	**1.26(1.02,1.57)**	0.02	**1.29(1.03,1.61)**	0.01	1.18(0.86,1.62)	0.01
**Ethnicity**										
Caucasians	6	1516/141	1.14(0.82,1.59)	0.08	1.21(0.95,1.55)	0.03	1.20 (0.95,1.52)	0.03	1.04(0.78,1.39)	0.15
others	3	374/353	2.07(0.72,5.98)	0.01	1.44(0.86,2.41)	0.10	1.56(0.85,2.85)	0.03	1.65(0.68,4.01)	0.02
**Source of controls**										
HCC^c^	4	683/752	1.30(0.73,2.29)	0.04	1.15(0.70,1.89)	0.01	1.20(0.77,1.86)	0.01	1.19(0.71,1.98)	0.06
PCC^c^	5	1207/1142	1.40(0.80,2.47)	0.00	**1.28(1.05,1.57)**	0.29	**1.34(1.02,1.77)**	0,04	1.18(0.75,1.88)	0.02
**Study sample size**										
≥500	2	709/708	0.96(0.66,1.39)	0.84	1.12(0.90,1.40)	0.66	1.09(0.89,1.35)	0.66	0.91(0.63,1.30)	0.93
<500	7	1181/1186	1.52(0.93,2.48)	0.00	1.32(0.97,1.80)	0.01	**1.37(1.01,1.87)**	0.00	1.30(0.86,1.97)	0.01
**Matched control**										
Yes	3	617/549	1.06(0.59,1.90)	0.06	1.46(0.82,2.58)	0.01	1.33(0.75,2.35)	0.00	0.89(0.64,1.24)	0.35
No	6	1273/1345	1.56(0.94,2.59)	0.01	1.17(0.96,1.42)	0.27	1.26(0.99,1.60)	0.07	1.40(0.91,2.17)	0.02
**HWE** ^**d**^ **in controls**										
Yes	7	1600/127	**1.50(1.00,2.23)**	0.01	**1.38(1.09,1.75)**	0.04	**1.42(1.12,1.80)**	0.02	1.27(0.90,1.79)	0.02
No	2	290/367	0.80(0.39,1.67)	0.23	0.88(0.62,1.24)	0.86	0.85(0.62,1.16)	0.70	0.84(0.40,1.76)	0.22

^a^Number of comparisons.

^b^P _value_ of Q-test for heterogeneity test. Random-effects model was used when P_value_ <0.1_,_ otherwise, fixed-effects model was adopted

^c^HCC, hospital-based case control; PCC, population-based case control.

^d^HWE: P for Hardy–Weinberg.

To determine the reason underlying the potential underestimation of the true effect of these polymorphisms on HNC risk, we performed subgroup analysis according to ethnicity, source of controls, study sample size, matched controls, and HWE in controls. Different ethnicities were categorized as Caucasians and others, while different sources of controls were defined as HCC and PCC.

Regarding ethnicity and the source of controls subgroup analysis, a significantly increased HNC risk was observed in PCC studies (*Tyr/His* vs. *Tyr/Tyr*, OR = 1.28, 95%CI = 1.05–1.57; *His/His*+ *Tyr/His* vs. *Tyr/Tyr*, OR = 1.34, 95%CI = 1.02–1.77). When stratified by study size, a significant association was found in studies with less than 500 participants (*His/His*+ *Tyr/His* vs. *Tyr/Tyr*, OR = 1.37, 95%CI = 1.01–1.87). This association remained consistently strong when the analyses were limited to studies in which genotype frequencies were in HWE (*His/His* vs. *Tyr/Tyr*, OR = 1.50, 95%CI = 1.00–2.23; *Tyr/His* vs. *Tyr/Tyr*, OR = 1.38, 95%CI = 1.09–1.75; *His/His*+ *Tyr/His* vs. *Tyr/Tyr*, OR = 1.42, 95%CI = 1.12–1.80) (Table 2).

#### The *EPHX1 His139Arg* polymorphism and HNC susceptibility

**Fig 3:**
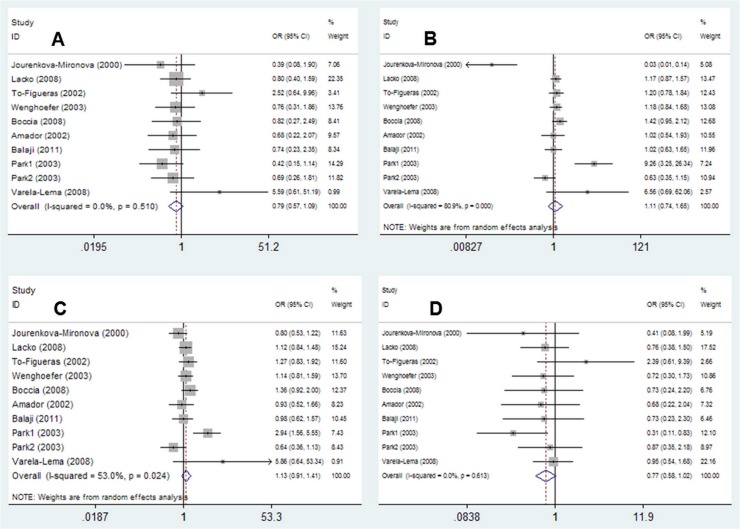
Forest plots of ORs with 95% CIs for *EPHX1 His139Arg* polymorphisms and HNC risk. The center of each square represents the OR, the area of the square is the number of sample and thus the weight used in the meta-analysis, and the horizontal line indicates the 95%CI. (A) *Arg/Arg* vs. *His/His*. (B) *Arg/His* vs. *His/His*. (C) *Arg/Arg*+*Arg/His* vs. *His/His*. (D) *Arg/Arg* vs.*Arg/His*+*His/His*.

Nine articles [24–32] were included in this meta-analysis that described 10 case-control studies, with 1982 cases and 2024 controls, reporting the association between the EPHX1 His139Arg polymorphism and HNC susceptibility. The main results of this pooled analysis are presented in Table 3 and Fig 3 shows forest plots illustrating the effect of the *EPHX1 Tyr113His* polymorphism on HNC risk.

**Table 3:** Quantitative analyses of the *EPHX1 His139Arg* polymorphism on the head and neck cancer risk.

Genetic model			Homozygote		Heterozygote		Dominant model		Recessive model	
Variables	Sample size		*Arg/Arg* vs. *His/His*		*Arg/His* vs. *His/His*		*Arg/Arg*+*Arg/His* vs. *His/His*		*Arg/Arg* vs.*Arg/His*+*His/His*	
	N^a^	Case/control	OR(95%CI)	*P*_*value*_ ^b^	OR(95%CI)	*P*_*value*_ ^b^	OR(95%CI)	*P*_*value*_ ^b^	OR(95%CI)	*P*_*value*_ ^b^
Total	10	1982/2024	0.79(0.57,1.09)	0.51	1.11(0.74,1.65)	0.00	1.13(0.91,1.41)	0.02	0.77(0.58,1.02)	0.60
**Ethnicity**										
Caucasians	7	1608/1671	0.83(0.56,1.21)	0.23	1.24(0.72,2.16)	0.00	1.27(0.98,1.64)	0.03	0.77(0.56,1.07)	0.30
others	3	374/353	0.70(0.38,1.30)	0.99	0.88(0.64,1.22)	0.42	0.86(0.63,1.16)	0.50	0.77(0.42,1.40)	0.94
**Source of controls**										
HCC^c^	4	683/752	0.69(0.41,1.15)	0.29	1.08(0.29,3.99)	0.00	1.28(0.73,2.25)	0.00	0.72(0.49,1.07)	0.34
PCC^c^	5	1299/1272	0.86(0.57,1.32)	0.61	1.14(0.96,1.37)	0.98	1.11(0.93,1.31)	0,91	0.83(0.55,1.26)	0.62
**Study sample size**										
≥500	2	709/708	0.78(0.46,1.35)	0.94	1.17(0.94,1.47)	0.95	1.12(0.90,1.40)	0.94	0.74(0.43,1.28)	0.92
<500	8	1273/1316	0.79(0.53,1.19)	0.31	1.08(0.58,1.98)	0.00	1.15(0.84,1.58)	0.01	0.78(0.56,1.09)	0.40
**Matched control**										
Yes	3	617/549	0.66(0.33,1.34)	0.74	0.46(0.12,1.80)	0.00	1.04(0.76,1.42)	0.19	0.64(0.32,1.29)	0.81
No	7	1365/1475	0.83(0.57,1.20)	0.28	1.32(0.89,1.97)	0.00	1.20(0.89,1.62)	0.02	0.80(0.59,1.09)	0.35

^a^Number of comparisons.

^b^P _value_ of Q-test for heterogeneity test. Random-effects model was used when P_value_ <0.1_,_ otherwise, fixed-effects model was adopted

^c^HCC, hospital-based case control; PCC, population-based case control.

Overall, the *EPHX1 His139Arg* polymorphism was not significantly associated with HNC susceptibility in four genetic models: *Arg/Arg*+ *Arg/His* vs. *His/His* (dominant model, OR = 1.13, 95% CI = 0.91–1.41), *Arg/Arg* vs. *Arg/His*+*His/His* (recessive model, OR = 0.77, 95% CI = 0.58–1.02), *Arg/Arg* vs. *His/His* (homozygote comparison, OR = 0.79, 95% CI = 0.57–1.09), and *Arg/His* vs. *His/His* (heterozygote comparison, OR = 1.11, 95% CI = 0.74–1.65). There were no significant associations found in the four genetic models between the *His139Arg* polymorphism and HNC susceptibility in any of the subgroup analyses (Table 3).

### Heterogeneity analysis

For the *Tyr113His* polymorphism, significant heterogeneity was found in the overall comparisons in four genetic models: dominant model P = 0.01, recessive model P = 0.01, homozygote comparison P = 0. 00, and heterozygote comparison P = 0.02.

Significant heterogeneity was also detected for the *His139Arg* polymorphism. No significant heterogeneity was found in the homozygote comparison or the recessive model comparison; however, significant heterogeneity was detected in the heterozygote comparison and dominant model (dominant model P = 0.02, recessive model P = 0.60, homozygote comparison P = 0.51, and heterozygote comparison P = 0.00.). Galbraith plot analyses were used to evaluate the potential sources of heterogeneity in this article. In this analysis, three studies [24, 28, 32] were found to be contributors of heterogeneity for the *Tyr113His* polymorphism (S1 Fig). After excluding these three outlier studies, we re-evaluated the association with reduced heterogeneity (*His/His* vs. *Tyr/Tyr*: P = 0.55; *Tyr/His* vs. *Tyr/Tyr*: P = 0.66; *His/His*+ *Tyr/His* vs. *Tyr/Tyr*: P = 0.86; *His/His* vs. *Tyr/His*+ *Tyr/Tyr*: P = 0.32). Regarding the *His139Arg* polymorphism, two studies [24, 31] were found to be contributors of heterogeneity (S1 Fig), and this heterogeneity was significantly reduced after the two studies were excluded(*Arg/Arg* vs. *His/His*: P = 0.58; *Arg/Arg* vs. *Arg/His*+ *His/His*: P = 0.89).

### Heterogeneity analysis

For the *Tyr113His* polymorphism, significant heterogeneity was found in the overall comparisons in four genetic models: dominant model P = 0.01, recessive model P = 0.01, homozygote comparison P = 0. 00, and heterozygote comparison P = 0.02.

Significant heterogeneity was also detected for the *His139Arg* polymorphism. No significant heterogeneity was found in the homozygote comparison or the recessive model comparison; however, significant heterogeneity was detected in the heterozygote comparison and dominant model (dominant model P = 0.02, recessive model P = 0.60, homozygote comparison P = 0.51, and heterozygote comparison P = 0.00.). Galbraith plot analyses were used to evaluate the potential sources of heterogeneity in this article. In this analysis, three studies [24, 28, 32] were found to be contributors of heterogeneity for the *Tyr113His* polymorphism (S1 Fig). After excluding these three outlier studies, we re-evaluated the association with reduced heterogeneity (*His/His* vs. *Tyr/Tyr*: P = 0.55; *Tyr/His* vs. *Tyr/Tyr*: P = 0.66; *His/His*+ *Tyr/His* vs. *Tyr/Tyr*: P = 0.86; *His/His* vs. *Tyr/His*+ *Tyr/Tyr*: P = 0.32). Regarding the *His139Arg* polymorphism, two studies [24, 31] were found to be contributors of heterogeneity (S1 Fig), and this heterogeneity was significantly reduced after the two studies were excluded (*Arg/Arg* vs. *His/His*: P = 0.58; *Arg/Arg* vs. *Arg /His*+ *His/His*: P = 0.89).

### Sensitivity analysis

Sensitivity analysis was conducted to detect the influence of a single study on the overall meta-analysis by omitting one study at a time. Regarding the association of the *EPHX1 Tyr113His* polymorphism with HNC risk, the study [32] seemed to have the greatest influence on the overall pooled estimates. Without that study, a re-evaluation of the meta-analysis showed that the OR became 1.13 (95% CI: 0.91–1.41). Compared with the previous result (OR = 1.35, 95% CI: 0.93–1.96), this result was not obviously different, which indicated the stability of the results. Regarding the association of the *EPHX1 His139Arg* polymorphism with CRC risk, the study [31] seemed to have the most influence on the overall pooled estimates. After the removal of this study, the meta-analysis showed that the OR changed from 1.11 (95% CI: 0.74–1.65) to 1.98 (95% CI: 0.84–1.14), which indicated the stability of the results. When the studies that were not in HWE were excluded, the estimated pooled OR was only altered in the homozygote comparison for the *Tyr113His* polymorphism (Table 2), demonstrating that our results were reliable.

### Cumulative meta-analysis

Cumulative meta-analyses were used to examine how the evidence has changed over time. In this article, we conducted cumulative meta-analyses of the 2 associations by categorizing the studies by publication time.

S2 Fig displays the results from the cumulative meta-analysis of the association between the *EPHX1 Tyr113His* polymorphism (*His/His*+ *Tyr/His* vs. *Tyr/Tyr*) and overall HNC in chronological order. The results showed a minimally significant association between the *EPHX1 Tyr113His* polymorphism and HNC risk when the data were categorized by publication year. A lack of significant associations between the *EPHX1 His139Arg* polymorphism and HNC risk was observed when the data were categorized by publication year (S2 Fig).

### Publication bias

Publication bias appears if no significant findings remain unpublished, which will artificially expand the apparent magnitude of an effect. In this meta-analysis, funnel plot, Begg’s and Egger’s tests were used to evaluate publication bias of the literature on HNC. S3 Fig and S4 Fig show funnel plots of overall HNC risk and *EPHX1* polymorphisms with basic symmetry, which suggested a lack of publication bias. Moreover, the results of statistical analysis revealed that publication bias was not evident, with the exception of the meta-analysis of the *EPHX1 His139Arg* homozygote comparison [(1) *EPHX1Tyr113His*, *His/His* vs. *Tyr/Tyr*: Begg’s test P = 0.12, Egger’s test P = 0.34; *Tyr/His* vs. *Tyr/Tyr*: Begg’s test P = 0.40, Egger’s test P = 0.25; dominant model: Begg’s test P = 0.18, Egger’s test P = 1.30; recessive model: Begg’s test P = 0.12, Egger’s test P = 0.46. (2) *EPHX1 His139Arg*, *Arg/Arg* vs. *His/His*: Begg’s test P = 0.28, Egger’s test P = 0.05; *Arg/His* vs. *His/His*: Begg’s test P = 0.86, Egger’s test P = 0.16; dominant model: Begg’s test P = 0.86, Egger’s test P = 0.61; recessive model: Begg’s test P = 0.86, Egger’s test P = 0.59](Table 4).

**Table 4:** Pooled data for *EPHX1*and HNC risk in meta-analyses.

		*EPHX1 Tyr113His*				*EPHX1 His139Arg*			
		*His/His* vs. *Tyr/Tyr*	*Tyr/His* vs. *Tyr/Tyr*	*His/His*+ *Tyr/His* vs. *Tyr/Tyr*	*His/His* vs. *Tyr/His* +*Tyr/Tyr*	*Arg/Arg* vs. *His/His*	*Arg/His* vs. *His/His*	*Arg/Arg*+*Arg/His* vs. *His/His*	*Arg/Arg* vs. *Arg/His*+*His/*His
P-Publication bias	Egger	0.34	0.25	1.30	0.46	0.05	0.16	0.61	0.59
	Begg	0.12	0.40	0.18	0.12	0.28	0.86	0.86	0.86
Heterogeneity test	P value	0.00	0.02	0.01	0.01	0.51	0.00	0.02	0.61
	I^2^	66.4%	55.1%	63.8%	58.2%	0%	80.9%	53.0%	0%

1. Fixed effects models were used, weighted by the inverse variance; 2. P < 0.1 is considered statistically significant for Q statistics; I^2^ is interpreted as the proportion of total variation contributed by between-study variation; 3. Egger’s test and Begg’s test to evaluate publication bias, P < 0.05 is considered

## Discussion

Some previous articles reported a significant association between *EPHX1*polymorphism and several human diseases, such as type 2 diabetes mellitus [36], alcohol dependence [37], and chronic obstructive pulmonary disease [38], while others found no such association [39–41]. In this meta-analysis, the pooled results indicated that subjects carrying the *EPHX1 Tyr113His* genotype had an increased risk of developing HNC. Our approach also allowed us to identify some potential differences, such as ethnicity, source of controls, study sample size and others.

In different ethnicities, the *EPHX1* polymorphism did not have a significant association with HNC risk. Our result differed from that of Zhong J H [42] and Liu F [11], which may be due to the limited number of studies in these analyses. Thus, well-designed studies with a large sample size that evaluate multiple ethnicities are required. The results of meta-analyses often depend on control selection procedures [43], such as the source of participants. In this meta-analysis, a statistically significant association between the *EPHX1 Tyr113His* polymorphism and HNC was observed in PCC studies, but not HCC studies, as the latter may contribute to some selection biases. Controls from a hospital population may not be a representative of the general population, especially when the investigated genotypes are associated with disease conditions [11]. In a subgroup analysis of controls in HWE, the 2 associations were only statistically significant in studies in which the controls followed HWE. Potential errors may occur in studies examining populations that deviate from HWE, such as laboratory or genotyping errors, population stratification, selection bias in the choice of controls and other confounding factors [44–45]. Simultaneously, the limited sample size may be an obstacle to obtaining accurate results. In this meta-analysis, a statistically significant association between the *EPHX1 Tyr113His* polymorphism and HNC was observed in studies enrolling less than 500 participants.

The degree of heterogeneity in a meta-analysis partially determines the difficulty in drawing overall conclusions [46]. Q-test and I^2^ statistics were calculated to test the impact of heterogeneity on the overall comparisons and some subgroup analyses where *P*<0.1 was considered significant heterogeneity and I^2^>50% indicated large heterogeneity. Galbraith plot analyses were used to evaluate the potential sources of heterogeneity in this article. In this analysis, three studies [24, 28, 32] were found to be contributors of heterogeneity for the Tyr113His polymorphism, while two studies [24, 31] were found to be contributors of heterogeneity for the His139Arg polymorphism. The heterogeneity was significant reduced after excluding these outlier studies; however, the conclusion was still consistent in the overall comparisons. Furthermore, although we conducted a sensitivity analysis by omitting one study at a time, we found no significant difference, indicating that our results were statistically reliable. Cumulative meta-analyses were also conducted to examine how the evidence has changed over time, and the results were borderline significant.

However, the meta-analysis described herein had some limitations. First, unadjusted OR estimates were adopted in this meta-analysis because we could not obtain sufficient information to calculate adjusted ORs with potential confounders, such as age and sex. Second, the study size and the sample size for some subgroup analyses were limited, which contributes to the possibility of type I and type II errors. Thus, the reliability of our results may need to be further tested. Third, similar to previous studies[11], this meta-analysis only focused on analyzing two individual *SNPs*—a combination (two-*SNP*) analysis was not performed. However, the activity of *EPHX1* can be affected by single *Tyr113His* and *His139Arg* polymorphisms or a combination of these polymorphisms [46–47]. In this analysis, only combination (two-*SNP*) analyses were considered, which prevented us from performing a pooled analysis. Finally, significant heterogeneity was found in studies examining *EPHX1* polymorphisms, and in some studies, the genotype distribution showed a deviation from HWE. As a retrospective study, the quality of a meta-analysis is dependent on the methodological limitations of the original studies. To minimize bias, we first designed a detailed protocol and performed a meticulous search as a predefined search strategy.

In conclusion, this meta-analysis evaluates the relationship between *EPHX1* polymorphisms and HNC risk. Our meta-analysis suggests that the *EPHX1 Tyr113His* polymorphism may be a risk factor for HNC, while the *EPHX1*

*His139Arg* polymorphism has no association with HNC risk. In subgroup analysis, a statistically significant association between the *EPHX1 Tyr113His* polymorphism and HNC was observed in population-based case-control studies, studies that enrolled more than 500 participants and studies in which the genotype frequencies were in HWE. When stratified by ethnicity, no significant associations were found in this study; thus, well-designed studies with a large sample size that examine multiple ethnicities are required. Moreover, further meta-analyses must be performed to estimate the effects of both single *SNP* analysis and combination (two-*SNP*) analysis. Gene–environment interactions should also be examined to determine the association between the *EPHX1* polymorphisms and HNC risk.

## Supporting Information

S1 PRISMA Checklist PRISMA checklist. (PDF)

S1 Fig Galbraith plots for *EPHX1* heterogeneity test of polymorphisms. A: *His/His*+ *Tyr/His* vs. *Tyr/Tyr*; B: *Arg/Arg*+ *Arg/His* vs. *His/Hi*s. The studies outside the range between -2 and 2 were seen as the outliers and the major source of heterogeneity.
(TIF)

S2 Fig Cumulative meta-analysis of associations between *EPHX1* polymorphisms and HNC risk. A. *His/His*+ *Tyr/His* vs. *Tyr/Tyr*; B. *Arg/Arg*+*Arg/His* vs. *His/His*.
(TIF)

S3 Fig Begg’s funnel plot for publication bias test. (A) For *EPHX1 Tyr113His* polymorphism.
(TIF)

S4 Fig Begg’s funnel plot for publication bias test. (B) For *EPHX1 His139Arg* polymorphism.
(TIF)

## References

[1] Marcu LG, Yeoh E (2009) A review of risk factors and genetic alterations in head and neck carcinogenesis and implications for current and future approaches to treatment. J Cancer Res Clin Oncol 135: 1303–1314. 10.1007/s00432-009-0648-7 19641938 PMC12160264

[2] Kamangar F, Dores GM, Anderson WF (2006) Patterns of cancer incidence, ortality, and prevalence across five continents: defining priorities to reduce cancer disparities in different geographic regions of the world. J Clin Oncol 24: 2137–2150. 16682732 10.1200/JCO.2005.05.2308

[3] Parkin DM, Pisani P, Ferlay J (1999) Global cancer statistics. CA Cancer J Clin 49: 33–64. 10.3322/canjclin.49.1.33 10200776

[4] Jemal A, Tiwari RC, Murray T, Ghafoor A, Samuels A, et al (2004) Cancer statistics, 2004. CA Cancer J Clin 54: 8–29. 10.3322/canjclin.54.1.8 14974761

[5] Hiyama T, Sato T, Yoshino K, Tsukuma H, Hanai A, et al (1992) Second primary cancer following laryngeal cancer with special reference to smoking habits. Jpn J Cancer Res 83: 334–339. 1506266 10.1111/j.1349-7006.1992.tb00111.x PMC5918829

[6] Geisler SA, Olshan AF (2001) GSTM1, GSTT1, and the risk of squamous cell carcinoma of the head and neck: a mini-HuGE review. Am J Epidemiol 154: 95–105. 11447041 10.1093/aje/154.2.95

[7] Pfeifer GP, Denissenko MF, Olivier M, Tretyakova N, Hecht SS, et al(2002) Tobacco smoke carcinogens, DNA damage and p53 mutations in smoking-associated cancers. Oncogene 21: 7435–7451. 12379884 10.1038/sj.onc.1205803

[8] Zhong Y, Carmella SG, Upadhyaya P, Hochalter JB, Rauch D, et al (2011) Immediate consequences of cigarette smoking: rapid formation of polyc yclic aromatic hydrocarbon diol epoxides. Chem Res Toxicol 24: 246–252. 10.1021/tx100345x 21184614 PMC3042042

[9] Hoeijmakers JH (2001) Genome maintenance mechanisms for preventing cancer. Nature 411: 366–374. 11357144 10.1038/35077232

[10] Arand M, Cronin A, Adamska M, Oesch F (2005) Epoxide hydrolases: structure, function, mechanism, and assay. Methods Enzymol 400: 569–588. 16399371 10.1016/S0076-6879(05)00032-7

[11] Liu F, Yuan D, Wei Y, et al (2012) Systematic review and meta-analysis of the relationship between EPHX1 polymorphisms and colorectal cancer risk. PloS one 7(8): e43821 10.1371/journal.pone.0043821 22928041 PMC3426545

[12] Beetham JK, Grant D, Arand M, et al (1995) Gene evolution of epoxide hydrolases and recommended nomenclature. DNA and Cell Biology, vol. 14, no. 1, pp. 61–71. 7832993 10.1089/dna.1995.14.61

[13] Fretland AJ, Omiecinski CJ (2000) “Epoxide hydrolases: biochemistry and molecular biology,” Chemico-Biological Interactions, vol. 129, no. 1–2, pp. 41–59. 11154734 10.1016/s0009-2797(00)00197-6

[14] Zhang JH, Jin X, Li Y, Wang R, Guo W, et al (2003) Epoxide hydrolase Tyr113His polymorphism is not associated with susceptibility to esophageal squamous cell carcinoma in population of North China. World J Gastroenterol 9: 2654–2657. 14669306 10.3748/wjg.v9.i12.2654 PMC4612025

[15] Hassett C, Aicher L, Sidhu JS, Omiecinski CJ (1994) Human microsomal epoxide hydrolase: genetic polymorphism and functional expression in vitro of amino acid variants. Hum Mol Genet 3: 421–428. 7516776 10.1093/hmg/3.3.421 PMC4868095

[16] Liu L, Liu L, Zeng F, Wang K, Huang J, et al (2011) Meta-analysis of the association between VEGF-634 G.C and risk of malignancy based on 23 casecontrol studies. J Cancer Res Clin Oncol 137: 1027–1036. 10.1007/s00432-010-0966-9 21174216 PMC11828216

[17] Attia J, Thakkinstian A, D’Este C (2003) Meta-analyses of molecular association studies: methodologic lessons for genetic epidemiology. J Clin Epidemiol 56: 297–303. 12767405 10.1016/s0895-4356(03)00011-8

[18] Cochran WG (1954) The combination of estimates from different experiments. Biometrics 10: 101–129.

[19] Higgins JP, Thompson SG, Deeks JJ, Altman DG (2003) Measuring inconsistency in meta- analyses. BMJ 327: 557–560. 12958120 10.1136/bmj.327.7414.557 PMC192859

[20] Mantel N, Haenszel W (1959) Statistical aspects of the analysis of data from retrospective studies of disease. J Natl Cancer Inst 22: 719–748. 13655060

[21] DerSimonian R, Laird N (1986) Meta-analysis in clinical trials.Control Clin Trials 7: 177–188. 3802833 10.1016/0197-2456(86)90046-2

[22] Begg CB, Mazumdar M (1994) Operating characteristics of a rank correlation test for publication bias. Biometrics 50: 1088–1101. 7786990

[23] Egger M, Davey Smith G, Schneider M, Minder C (1997) Bias in meta-analysis detected by a simple, graphical test. BMJ 315: 629–634. 9310563 10.1136/bmj.315.7109.629 PMC2127453

[24] Jourenkova-Mironova N, Mitrunen K, Bouchardy C, et al (2000) High-activity microsomal epoxide hydrolase genotypes and the risk of oral, pharynx, and larynx cancers. Cancer research 60(3): 534–536. 10676631

[25] Lacko M, Roelofs HMJ, te Morsche RHM, et al (2008) Microsomal epoxide hydrolase genotypes and the risk for head and neck cancer[J]. Head & neck 30(7): 836–844. 18383527 10.1002/hed.20781

[26] To-Figueras J, Gené M, Gómez-Catalán J, et al (2002) Microsomal epoxide hydrolase and glutathione S-transferase polymorphisms in relation to laryngeal carcinoma risk. Cancer letters 187(1): 95–101. 12359356 10.1016/s0304-3835(02)00406-8

[27] Wenghoefer M, Pesch B, Harth V, et al (2003) Association between head and neck cancer and microsomal epoxide hydrolase genotypes. Archives of toxicology 77(1): 37–41. 12491039 10.1007/s00204-002-0414-y

[28] Boccia S, Cadoni G, Sayed-Tabatabaei FA, et al (2008) CYP1A1, CYP2E1, GSTM1, GSTT1, EPHX1 exons 3 and 4, and NAT2 polymorphisms, smoking, consumption of alcohol and fruit and vegetables and risk of head and neck cancer. Journal of cancer research and clinical oncology 134(1): 93–100. 17611777 10.1007/s00432-007-0254-5 PMC12161748

[29] Balaji L, Lakkakula BVKS, Krishna BS, et al (2011) Lack of association of EPHX1 genotypes and haplotypes with oral cancer in South Indians. Genetic testing and molecular biomarkers 15(9): 595–599. 10.1089/gtmb.2010.0260 21453055

[30] Varela-Lema L, Ruano-Ravina A, Juiz Crespo MA, et al (2008) CYP1A1, mEH, and GSTM1 polymophisms and risk of oral and pharyngeal cancer: a Spanish case-control study. Journal of oncology 2008: 741310 10.1155/2008/741310 19259333 PMC2648631

[31] Park JY, Schantz SP, Lazarus P (2003) Epoxide hydrolase genotype and orolaryngeal cancer risk: interaction with GSTM1 genotype. Oral oncology,39(5): 483–490. 12747973 10.1016/s1368-8375(03)00008-3 PMC3715071

[32] Amador AG, Righi PD, Radpour S, Everett ET, et al (2002)Polymorphisms of xenobiotic metabolizing genes in oropharyngeal carcinoma. Oral Surg Oral Med Oral Pathol Oral Radiol Endod 93: 440–445. 12029283 10.1067/moe.2002.122586

[33] Lacko M, Voogd AC, Roelofs HMJ, et al (2013) Combined effect of genetic polymorphisms in phase I and II biotransformation enzymes on head and neck cancer risk. Head & neck 35(6): 858–867. 22715028 10.1002/hed.23054

[34] Janot F, Massaad L, Ribrag V, et al (1993) Principal xenobiotic-metabolizing enzyme systems in human head and neck squamous cell carcinoma Carcinogenesis 14(7): 1279–1283. 8330340 10.1093/carcin/14.7.1279

[35] Buch C, Nazar-Stewart V, Weissfeld JL, Romkes M (2008) Case–control study of oral and oropharyngeal cancer in whites and genetic variation in eight metabolic enzymes. Head & neck 30(9): 1139–1147. 18642288 10.1002/hed.20867 PMC3627181

[36] Ghattas MH, Amer MA (2012) Possible role of microsomal epoxide hydrolase gene polymorphism as a risk factor for developing insulin resistance and type 2 diabetes mellitus. Endocrine [Epub ahead of print]. 10.1007/s12020-012-9656-5 22555758

[37] Bhaskar LV, Thangaraj K, Patel M, Shah AM, Gopal K, et al. (2012) EPHX1 Gene Polymorphisms in Alcohol Dependence and their Distribution among the Indian Populations. Am J Drug Alcohol Abuse [Epub ahead of print]. 10.3109/00952990.2011.643991 22257321

[38] Chen CZ, Wang RH, Lee CH, Lin CC, Chang HY, et al (2011) Polymorphism of microsomal epoxide hydrolase is associated with chronic obstructive pulmonary disease and bronchodilator response. J Formos Med Assoc 110: 685–689. 10.1016/j.jfma.2011.09.003 22118311

[39] Dura P, Bregitha CV, Te Morsche RH, Roelofs HM, Kristinsson JO, et al (2012) EPHX1 polymorphisms do not modify esophageal carcinoma susceptibility in Dutch Caucasians. Oncol Rep 27: 1710–1716. 10.3892/or.2012.1734 22447130

[40] Nisa H, Budhathoki S, Morita M, Toyomura K, Nagano J, et al. (2012) Microsomal epoxide hydrolase polymorphisms, cigarette smoking, and risk of colorectal cancer: The Fukuoka Colorectal Cancer Study. Mol Carcinog [Epub ahead of print]. 10.1002/mc.21897 22415791

[41] Zhao ZQ, Guan QK, Yang FY, Zhao P, Zhou B, et al (2012) System review and metaanalysis of the relationships between five metabolic gene polymorphisms and colorectal adenoma risk. Tumour Biol 33(2): 523–535. 10.1007/s13277-011-0287-x 22161138

[42] Zhong JH, Xiang BD, Ma L, et al (2013) Meta-analysis of microsomal epoxide hydrolase gene polymorphism and risk of hepatocellular carcinoma. PloS one 8(2): e57064 10.1371/journal.pone.0057064 23451147 PMC3581564

[43] Benhamou S, Lee WJ, Alexandrie AK, Boffetta P, Bouchardy C, et al (2002) Meta- and pooled analyses of the effects of glutathione S-transferase M1 polymorphisms and smoking on lung cancer risk. Carcinogenesis 23: 1343–1350. 12151353 10.1093/carcin/23.8.1343

[44] Knechtel G, Szkandera J, Stotz M, Hofmann G, et al (2010) Single nucleotide polymorphisms in the hypoxia-inducible factor-1 gene and colorectal cancer risk. Mol Carcinog 49(9):805–809. 10.1002/mc.20655 20572162

[45] Min JH, Yang H, Ivan M, Gertler F, Kaelin WG Jr, Pavletich NP (2002) Structure of an HIF-1alpha-pVHL complex: hydroxyproline recognition in signaling. Science 296(5574):1886–1889. 12004076 10.1126/science.1073440

[46] Higgins J, Thompson SG (2001) Quantifying heterogeneity in a meta-analysis. Statistics in medicine 21(11): 1539–1558. 10.1002/sim.1186 12111919

[47] Hassett C, Lin J, Carty CL, Laurenzana EM, Omiecinski CJ (1997) Human hepatic microsomal epoxide hydrolase: comparative analysis of polymorphic expression. Arch Biochem Biophys 337: 275–283. 9016823 10.1006/abbi.1996.9794

