# Supplementary figures and images for: Systematic Review and Meta-Analysis of the Relationship between EPHX1 Polymorphisms and the Risk of Head and Neck Cancer
Source: PLoS One. 2015 Apr 29;10(4):e0123347. doi: 10.1371/journal.pone.0123347 (PMC4414537; doi:10.1371/journal.pone.0123347)

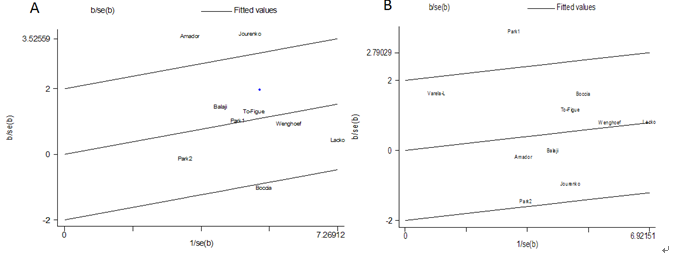

Supplement: S1 Fig — A: His/His+ Tyr/His vs. Tyr/Tyr; B: Arg/Arg+ Arg/His vs. His/His. The studies outside the range between -2 and 2 were seen as the outliers and the major source of heterogeneity. (TIF) [file pone.0123347.s002.tif]

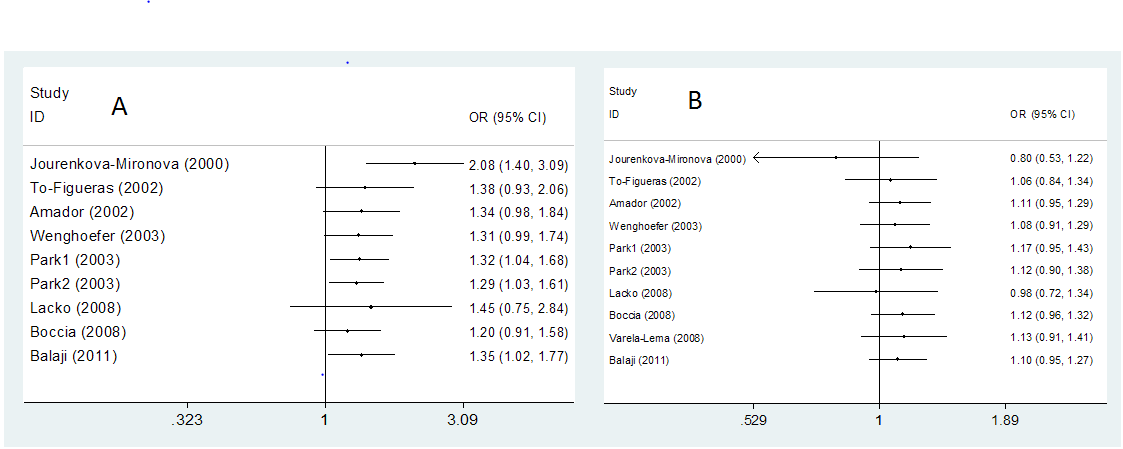

Supplement: S2 Fig — A. His/His+ Tyr/His vs. Tyr/Tyr; B. Arg/Arg+Arg/His vs. His/His. (TIF) [file pone.0123347.s003.tif]

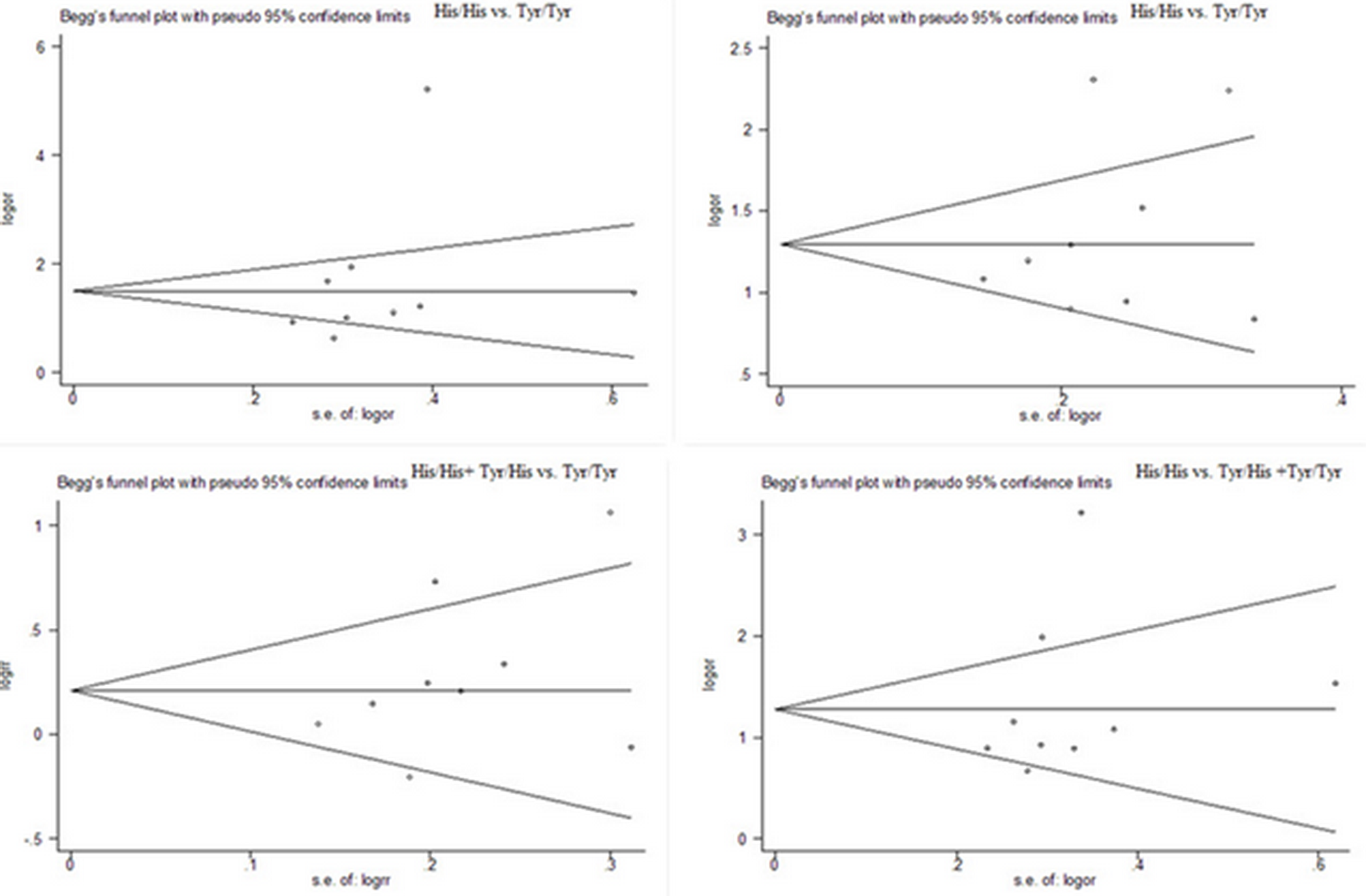

Supplement: S3 Fig — (A) For EPHX1 Tyr113His polymorphism. (TIF) [file pone.0123347.s004.tif]

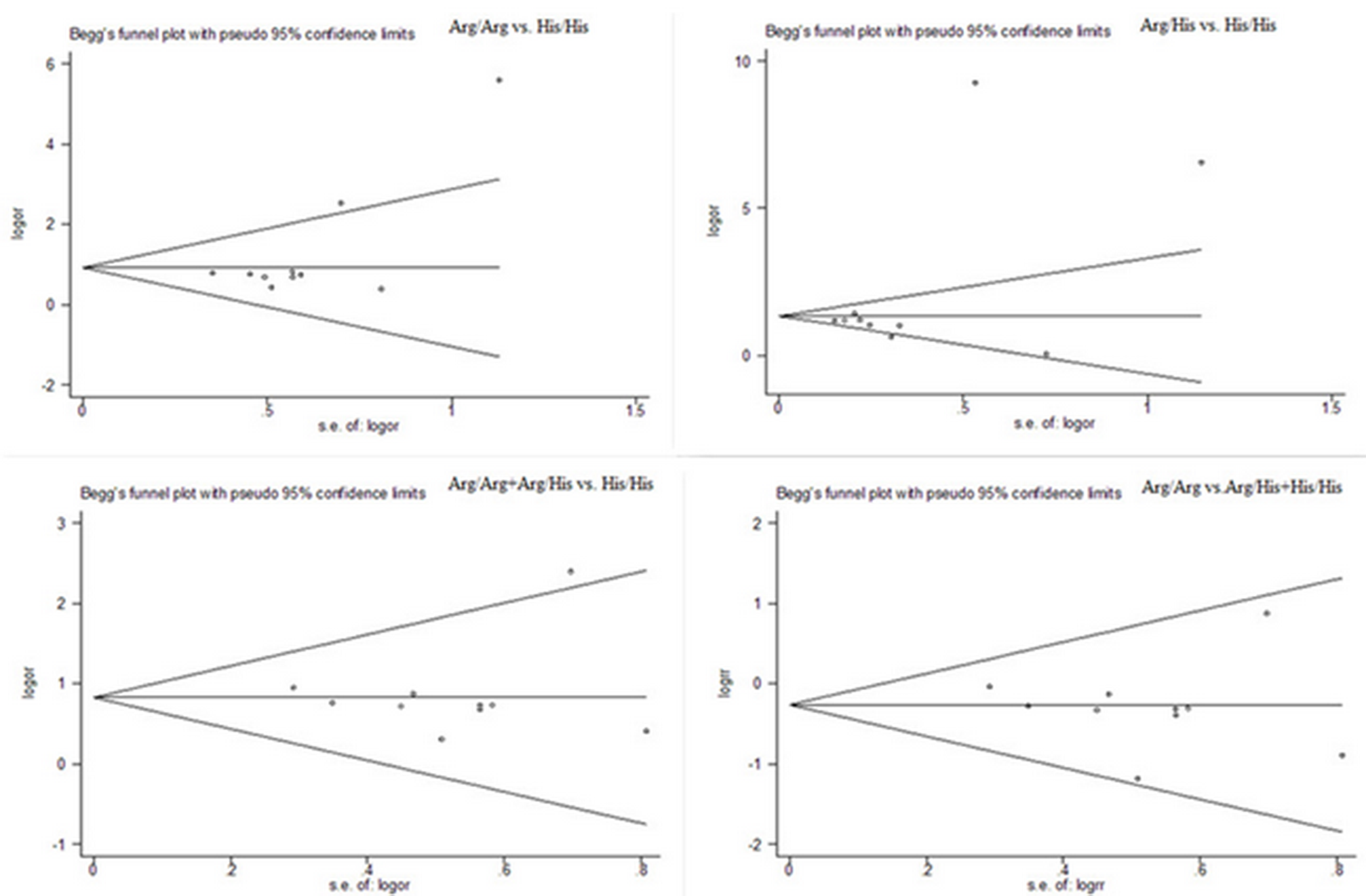

Supplement: S4 Fig — (B) For EPHX1 His139Arg polymorphism. (TIF) [file pone.0123347.s005.tif]
